# Enantioselective Liquid Chromatographic Separations Using Macrocyclic Glycopeptide-Based Chiral Selectors

**DOI:** 10.3390/molecules26113380

**Published:** 2021-06-03

**Authors:** Róbert Berkecz, Dániel Tanács, Antal Péter, István Ilisz

**Affiliations:** Interdisciplinary Excellence Centre, Institute of Pharmaceutical Analysis, University of Szeged, Somogyi u. 4, H-6720 Szeged, Hungary; berkecz.robert@szte.hu (R.B.); tanacsd95@gmail.com (D.T.); apeter@chem.u-szeged.hu (A.P.)

**Keywords:** enantiomer separations, chiral stationary phases, macrocyclic glycopeptide antibiotics, liquid chromatography

## Abstract

Numerous chemical compounds of high practical importance, such as drugs, fertilizers, and food additives are being commercialized as racemic mixtures, although in most cases only one of the isomers possesses the desirable properties. As our understanding of the biological actions of chiral compounds has improved, the investigation of the pharmacological and toxicological properties has become more and more important. Chirality has become a major issue in the pharmaceutical industry; therefore, there is a continuous demand to extend the available analytical methods for enantiomeric separations and enhance their efficiency. Direct liquid chromatography methods based on the application of chiral stationary phases have become a very sophisticated field of enantiomeric separations by now. Hundreds of chiral stationary phases have been commercialized so far. Among these, macrocyclic glycopeptide-based chiral selectors have proved to be an exceptionally useful class of chiral selectors for the separation of enantiomers of biological and pharmacological importance. This review focuses on direct liquid chromatography-based enantiomer separations, applying macrocyclic glycopeptide-based chiral selectors. Special attention is paid to the characterization of the physico-chemical properties of these macrocyclic glycopeptide antibiotics providing detailed information on their applications published recently.

## 1. Introduction

Nowadays, we already know that chirality, the most important form of molecular asymmetry, is universal, and chirality at the molecular level plays an essential role in biological systems. If a racemate pharmacon enters a living organism, its enantiomers may differ in their utilization, distribution, metabolism, thus in the type and scale of their biological effect. This is the reason why the pharmaceutical industry pays outstanding attention to chiral compounds when developing biologically active chiral pharmacons. It is important to realize that, as well as in pharmaceutical drugs, chiral products can be found in a vast number among food additives, agricultural chemicals, or fragrance materials of perfumes, where the quantitation of each enantiomer can also have a practical interest.

For the efficient separations of chiral compounds, techniques based on liquid chromatography (LC) employing chiral stationary phases (CSPs) are the most frequently applied solutions nowadays. Of the tremendous number of CSPs, the most frequently used chiral selectors are amino acids, proteins, derivatized linear or branched carbohydrates (e.g., cellulose or amylose), and cavity-type selectors, such as chiral crown ethers, cyclodextrins, cyclofructans, and macrocyclic antibiotics. These selectors and the CSPs made of them have been discussed in several reviews, books, and book chapters [1,2,3,4,5,6,7]. In the discussion of this review article, we focus on scientific results published only between 2015 and the first quarter of 2021 obtained with LC applying macrocyclic antibiotics as CSPs. For earlier publications of this topic, the reader should refer to several comprehensive reviews [8,9,10,11,12,13].

The use of macrocyclic antibiotics as chiral selectors was first described in 1994 by Armstrong et al. [14,15]. Thanks to intensive development efforts, robust, widely applicable stationary phases have been produced and commercialized in a short time under the trademark Chirobiotic^TM^ by Astec, and later by Sigma–Aldrich. The popularity of macrocyclic glycopeptide-based CSPs gained in recent years can be attributed primarily to the ability of antibiotics used as selectors to form different interactions in a variety of qualities and strengths. Unlike other selectors, this family includes hundreds of molecules bearing very diverse structures and rather different chemical properties; however, only a few of them appear to be effective as CSP. In general, their representatives have a molar mass between 600 and 2200 g mol^−1^. There are acidic, basic, and neutral compounds among them, and they are capable of forming a variety of interactions (e.g., electrostatic, hydrophobic-hydrophobic, π–π, H-bridge, steric hindrance, etc.). Due to their complex structure and multiple functional groups, a wide range of compounds can be enantioseparated. Because of the long-term stability, good efficiency, good loadability, and high reproducibility of the commercially available columns, these phases gained an important role in enantiomer separations.

Today, the stationary phase of the commercially available Chirobiotic columns is a macrocyclic glycopeptide chemically bound to silica gel. Another aspect that has become increasingly important in recent years in the selection of an LC column is that the separation system can be coupled to mass spectrometric (MS) detection. These columns meet this condition perfectly, as they can be operated with high efficiency in polar ionic (PI), and polar organic (PO) mode, and can also be applied under reversed-phase (RP) and normal-phase (NP) conditions, that is, they are multimodal. It is important to point out that changes in the chromatographic modes may lead to significantly different enantioselectivities due to the already mentioned structural variability of the antibiotic selectors. As a result, different mechanisms may dominate the separation mechanism by changing the composition of the mobile phase. This also creates additional opportunities for method development.

One of the most important features of antibiotic-based selectors is their ionic property. Ionic and ionizable functional groups can play an important role in the chiral recognition process. Therefore, knowing the structure of the enantiomers to be separated, the correct choice of column and chromatographic mode greatly speeds up the method development process. Chirobiotic columns very often show complementary properties to each other. Namely, if partial separation is achieved with one column, there is a good chance that baseline separation can be obtained on another Chirobiotic column. This property can be explained by the structural analogies of these CSPs. A characteristic feature of these CSPs is that their selector has a peptide backbone that allows H-bonding and dipole–dipole interactions to be formed. When separating ionic compounds, the ionizable functional groups (amino and/or carboxyl groups) naturally offer the possibility of ionic interactions. If the selector contains sugar units as well, these may play a role in the formation of additional H-bonds or, with their spatial location, may help or inhibit the enantiomeric recognition. Finally, it should be kept in mind that macrocycles can provide a basket-like structure; that is, inclusion complexation can occur under RP conditions (as observed frequently for cyclodextrins).

The very intense development of achiral stationary phases observed in the last decades has a significant impact on the evolution of CSPs. In addition to the physical dimensions of a column, the average size of the particles constituting the packing bed has a determining role in separation efficiency. In the case of commercially available CSPs, manufacturers are gradually moving from 5 µm to 3 µm particles. Further reducing the particle size may offer the possibility for very fast enantiomeric separations, high-throughput screenings, easier coupling of the chiral and achiral columns in a 2D-chromatographic system, or even for real-time monitoring of enantiomeric ratios in asymmetric syntheses. Depending on the physical parameters of the column and eluent viscosity, however, the full potential of high-efficiency particles can only be exploited on chromatographic hardware specialized to ensure elevated pressures and low dead volumes (ultrahigh-performance liquid chromatography, UHPLC). In a race for extremely fast enantiomeric separations, Armstrong and co-workers [16,17], Gasparrini and co-workers [18,19], and the Chankvetadze group [20,21] play a pioneering role in the development of CSPs based on superficially porous (SP) or fully porous (FP) particles. We believe that the development and application of columns, packed with highly efficient particles utilizing the selectors already proved their wide applicability, will be the most challenging area in “chiral chromatography” for the near future. It is expected that further developments of LC techniques will strengthen the dominant role of these CSPs, including macrocyclic glycopeptides.

In the following, we briefly discuss the structural characteristics of the most important macrocyclic antibiotics applied as CSPs.

## 2. Structural Characterization of the Most Important Antibiotics

**Figure 1:**
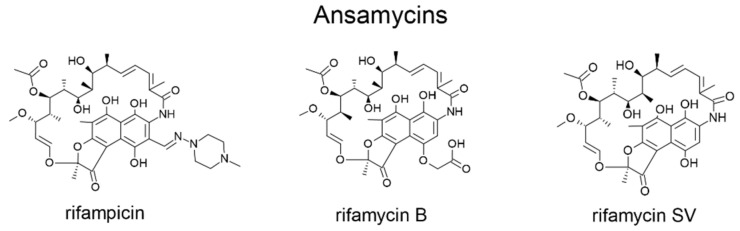
Structure of ansamycins.

**Figure 2:**
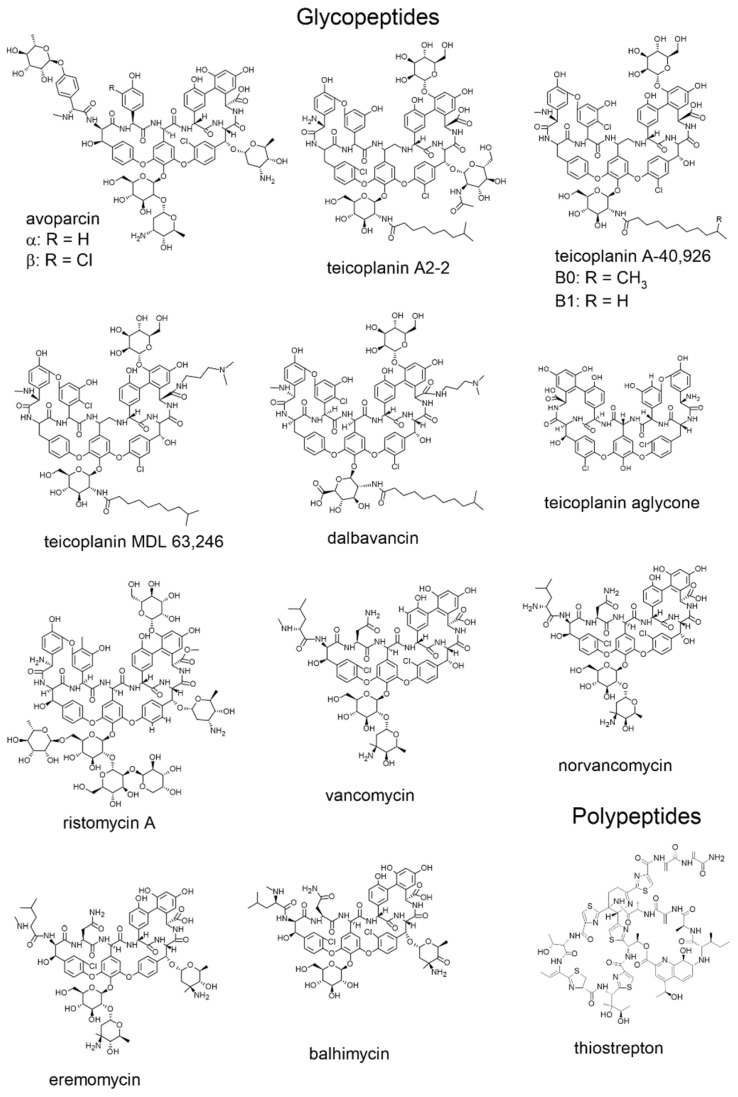
Structure of glycopeptides and polypeptides.

Macrocyclic antibiotics employed for chiral separations in LC include ansamycins (rifamycins, rifampicins), glycopeptides (avoparcin, teicoplanin, teicoplanin aglycon, ristocetin A, vancomycin and their analogs (dalbavancin, eremomycin, balhymicin)), and the polypeptide antibiotic thiostrepton. Some physical and chemical characteristics of the major representatives of antibiotics used as selectors in LC are shown in Table 1 and Table 2, while their structures are illustrated in Figure 1 and Figure 2. 

The most important representatives of the antibiotics applied as CSPs are vancomycin, ristocetin A, teicoplanin, and teicoplanin aglycon. Related pieces of information are discussed in the following subsections.

### 2.1. Vancomycin

Natural vancomycin (Figure 2) is produced by the bacterium *Streptomyces orientalis* [22]. It has a molar mass of 1449 g mol^−1^, and 18 stereogenic centers are located in the molecule. Vancomycin is highly soluble in water, indicating its polar character. It is composed of three macrocyclic components, which together form a basket-like structure with an apolar interior. This structure is also responsible for the formation of hydrophobic–hydrophobic interactions and steric effects. These, in many cases, play a key role in enantioselectivity, with an additional contribution from the two sugar units of vancomycin. The five aromatic rings are responsible for the formation of π–π bonds, while the π-acidic nature of the aromatic ring containing two chlorine substituents contributes mainly to the chiral recognition in NP mode. Two primary amino groups, a secondary amino group, and a carboxyl group are involved in ionic interactions.

### 2.2. Ristocetin A

Ristocetin A, with a molar mass of 2066 g mol^−1^ and 38 chirality centers (Figure 2), is the fermentation product of *Nocardia lurida* [23]. Ristocetin A is the most polar selector due to its 21 hydroxyl group, which is further strengthened by the lack of a hydrophobic nonyl chain. Similar to vancomycin, it contains an aglycone with a basket-like structure created by four macrocycles. A significant difference, however, is that there is no free carboxyl group. Instead, the molecule contains a methyl ester group, which may result in a weaker interaction with cationic compounds.

### 2.3. Teicoplanin and Teicoplanin Aglycon

Teicoplanin is a macrocyclic glycopeptide produced by the bacterium *Actinoplanes teichomyceticus*, a mixture of five molecules with very similar structures [24]. Of these, teicoplanin A_2–2_, produced in the largest amount (Figure 2), serves as the basis of the Chirobiotic T and T2 columns; therefore, we briefly discuss its structural characteristics. The basket-like structure similar to that of vancomycin is an important structural feature but, in this case, it is created by four macrocycles. The seven aromatic rings, two of which contain a chlorine substituent each, can participate in the formation of π–π bonds and π-acid, π-base interactions. A carboxyl (pK ~ 2.5) and a primary amino group (pK ~ 9.2) are responsible for the ionic nature of teicoplanin. It is important to note that the aglycon backbone of teicoplanin is associated with three sugar moieties—two d-glucosamines, and one d-mannose—and the nonyl chain responsible for the hydrophobic–hydrophobic interaction is attached to one of the d-glucosamine units. The possible contribution of the sugar moiety to the chiral recognition process may be realized in three ways [25]:

1. it may block access to the inside of the basket,
2. it may inhibit the possible interactions with the two phenolic and one alcoholic hydroxyl groups of the aglycon, through which the three sugar moieties are linked in the case of native teicoplanin,
3. the alcoholic hydroxyl, ether, and amide groups of the sugar moiety as well as the nonyl chain may provide additional interactions.

## 3. Retention Mechanism

Similar to achiral chromatography, in chiral chromatography upon selecting the appropriate CSP, retention and selectivity can be controlled through mobile phase composition. Glycopeptide-based CSPs, due to various functionalities in their structures, offer the possibility to be operated in different chromatographic modes. The peptide backbone provides hydrogen bonding and dipole–dipole interactions, depending on the pH and the ionizability of the analytes. In addition, the ionic sites offer the possibility for ionic interactions, while the sugar units, when present, may provide further hydrogen bonding and some steric effects. It is worth mentioning that, under RP conditions, internal ring structures facilitate inclusion complexation. Obviously, not all of these interactions are active for the retention and enantiodiscrimination in all mobile phases. However, varying the eluent composition, the availability and role of each interaction can be modulated, to achieve efficient separation. Protocols for the method development and detailed discussions on the retention mechanisms under different chromatographic conditions using macrocyclic glycopeptide CSPs have been published earlier by our group and others [8,9,10,11,12,13].

## 4. Recent Applications of Different Macrocyclic Antibiotic-Based CSPs

### 4.1. High-Performance Liquid Chromatographic Enantioseparation of Stereoisomers of Different Analytes on Vancomycin-Based CSPs

Vancomycin was the first macrocyclic antibiotic used as a stationary phase in chiral chromatography [14,15]. Since then, a number of research articles have described its effectiveness in the enantiomeric separation of different kinds of analytes, including amino acids and their derivatives as well as primary amines and drugs. Results published recently are summarized in Table 3.

Separation of enantiomers of trantinterol was carried out on Chirobiotic^TM^ V using MeOH/MeCN/AcOH/NH_4_OH mobile phases [26]. The optimized method was applied to determine the concentration of enantiomers in human plasma. It was observed that the concentration of (−)-trantinterol was higher after oral administration than that of (+)-trantinterol. Several amphetamine-type stimulants are widely abused as drugs, and different techniques have been utilized for their analysis so far. At first, these were non-chiral techniques, but recently, due to the need for enantioseparation, new enantioselective methods have been described. A rapid method was developed for the chiral separation and LS-MS/MS determination of amphetamine and methamphetamine from urine [27]. Separations were achieved on Chirobiotic^TM^ V2 with a MeOH-based mobile phase containing 0.1% glacial acetic acid and 0.02% ammonium hydroxide. 

Chirobiotic^TM^ V2 was applied in a study of five different clandestine drug laboratory samples [28]. Except for the enantiomers of pseudoephedrine, all amphetamine-type stimulants could be separated with a mobile phase based on MeOH and ammonium trifluoroacetic acid as an additive. Vancomycin was used as a chiral mobile phase additive to separate the enantiomers of ketoprofen using an achiral NH_2_ column [29]. The results obtained with phosphate buffer/2-propanol eluent showed that good resolution and selectivity were achievable, even at low vancomycin concentrations of 1–2 mM. Two CSPs obtained by the immobilization of crystalline degradation products of vancomycin on silica support were successfully applied in the enantioseparation of some acidic and basic drugs (amlodipine, atropine, baclofen, ibuprofen, mandelic acid, Phe) [30]. This result showed that synthetically modified vancomycin can also be used for enantiomeric separations. From a theoretical point of view, it is possible to mix different chiral selectors and gain the advantages of both. 

A mixed chiral sorbent containing eremomycin and vancomycin was synthesized and successfully employed for the enantiomeric separation of amino acids and β-blockers [31]. It was shown that vancomycin by itself was not able to separate the enantiomers of non-derivatized amino acids, while eremomycin could not separate the enantiomers of β-blockers. The separation ability of a sorbent based on silica, modified with gold nanoparticles and immobilized vancomycin, was also studied [32]. The new sorbent showed good efficiencies and reduced analysis times in the separation of β-blocker enantiomers. Vancomycin was bonded to silica gel through a carboxylic acid linker made with succinic anhydride [33]. The functionalization of the silica surface was characterized, and the newly synthesized CSP was successfully applied for the separation of mandelic acid enantiomers in NP mode using *n*-heptane/2-propanol/TFA. 

An exhaustive study on the enantiomeric separation of xanthone derivatives was carried out with Chirobiotic^TM^ V, T, TAG, and R columns in four chromatographic elution modes (NP, PO, PI, and RP mode) [34]. The xanthone derivatives could be separated with good selectivities and resolutions on at least one column, while the docking study showed different binding patterns for each selector due to their complex structures. The enantioseparation and adsorption thermodynamics of aromatic hydroxy acids and their derivatives on Nautilus-E, Nautilus-R (Biokhimmak, Moscow, Russia), and Chirobiotic T was investigated [35]. Different retention and enantiorecognition mechanisms on eremomycin and ristomycin were found compared to teicoplanin. Organic–inorganic hybrid monolithic columns, prepared using carbamoylated derivatives of erythromycin [36] and azithromycin [37], were tested for the enantioresolution of chiral drugs. Utilizing capillary electrochromatography, baseline resolution was achieved for six basic [36] and six acidic [37] drug enantiomers.

### 4.2. High-Performance Liquid Chromatographic Enantioseparation of Stereoisomers of Different Analytes on Teicoplanin, Teicoplanin Aglycon, Vancomycin, and Ristocetin A-Based CSPs

Of the macrocyclic glycopeptide-based CSPs, teicoplanin and its analogs have been employed most frequently for the enantioseparation of various compounds. Teicoplanin-based CSPs provide excellent enantioselectivity towards chiral amino acids [38]. Among others, amino acids and their analogs, drugs, and small peptides were recently enantioseparated, as summarized in Table 4. The commercially available columns are the teicoplanin-based Chirobiotic^TM^ T and Chirobiotic^TM^ T2 differing in their binding chemistry, the teicoplanin aglycone-based Chirobiotic^TM^ TAG, and the ristocetin A-based Chirobiotic^TM^ R, all immobilized on silica gel.

Enantiomers of four unnatural paclitaxel precursor phenylisoserine analogs were separated on Chirobiotic^TM^ T, TAG, and V CSPs in RP and PI modes [39]. Separation was found to be influenced by both the eluent pH and the MeOH content of 0.1% TEAA (pH 4.1)/MeOH mobile phase, where a different retention mechanism was suggested to interpret the retention behavior observed at high MeOH contents. Lehotay et al. [40,41] investigated the enantioseparation of Cys, homo-Cys, and Met as standards, and in human plasma with 2D-HPLC technique applying a Purospher C18 in the first and Chirobiotic^TM^ T or TAG in the second dimension. Separations were optimized using RP conditions, where amino acids were separated in the first, while their enantiomers in the second dimension. The effect of temperature on chromatographic parameters was investigated for the same system (Cys, homo-Cys, and Met) and thermodynamic parameters were calculated [42]. The applied RP mobile phases in all cases contained phosphate buffer and octanesulfonic acid, an ion-pairing reagent. Separation of therapeutic peptides was studied on Chirobiotic^TM^ T and V, three cyclofructane-based (CF-6), and a zwitterionic column applying hydrophilic interaction liquid chromatography (HILIC) conditions [43]. The separation performance of the columns studied was compared, and macrocyclic antibiotic-based CSPs were found to function well in both HILIC and RP modes, with hydrophilicity and ion exchange characteristics similar to other zwitterionic CSPs. 

Carbocyclic β-amino acids possessing limonene skeleton were enantioseparated on Chirobiotic^TM^ T, TAG, and R columns under RP and PI conditions [44]. A thermodynamic study showed a rather unusual entropically-driven separation in most cases, while the importance of the ionic interactions was validated by the simple displacement model. Enantiomers of Phe were separated with different types of CSPs under RP, NP and PO conditions [45]. The optimized HPLC-UV method employing Chirobiotic T was validated and applied for the quantitative determination of the enantiomeric composition of some energy drinks and dietary supplements.

The separation of enantiomers of ofloxacin was optimized by applying Chirobiotic^TM^ T, TAG, and R columns under RP and PI conditions [46]. The optimized method utilizing Chirobiotic^TM^ R was validated and applied for monitoring the enantioselective biodegradation of ofloxacin in activated sludge. Chromatographic resolution of the epimeric mixtures of fortimicin aminoglycosides was achieved in PI mode applying Chirobiotic^TM^ T [47]. As a result, ten natural fortimicin pseudodisaccharide analogs were identified and semi-quantified. Enantiomeric separation of racemic mixtures of citalopram analogs was performed by columns based on cyclodextrin and macrocyclic glycopeptide (Chirobiotic^TM^ T, TAG, R, V, and V2) [48]. PI, PO, and RP modes were tested, and vancomycin-based CSPs operated in PI mode were found to be the most effective in achieving baseline separation. The Chirobiotic^TM^ T column was utilized to the resolution of enantiomers of modafinil tested under NP, PO, and PI modes [49]. The best separation performance was found with the use of the MeOH/TEA mobile phase, where the addition of TEA resulted in enhanced reproducibility. The method was validated and successfully applied for the determination of modafinil enantiomers in pharmaceutical formulations. 

An enantioselective LC-MS/MS method was developed for the analysis of pharmacologically active compounds in environmental samples [50]. The method utilizing Chirobiotic^TM^ T in PI mode was successfully applied for monitoring metabolites of ibuprofen and other active pharmaceutical ingredients in influent and effluent wastewater and in river water. To improve the enantioseparation of acidic compounds (mandelic acid, vanylmandelic acid, phenyllactic acid), (1*S*)-(−)-borneol as well as (1*R*)-(+)-fenchol- [51] and menthol-based [52] chiral ionic liquids (CILs) as mobile phase additives were applied together with a Chirobiotic^TM^ T column in RP mode. The chiral salts exhibited a synergistic effect with the teicoplanin-based CSP, enhancing the resolution of acidic enantiomers. Structural task-specific properties of the new terpene-based chiral ionic liquids were confirmed by molecular modeling and docking simulations. Two commercially available columns, namely Chirobiotic^TM^ T and Nautilus-E (with eremomycin as a chiral selector) were tested for the enantiomeric purity control of albuterol [53]. After method optimization, Chirobiotic^TM^ T applied in PI mode offered the expected selectivity, and the enantiomeric purity could be measured in two pharmaceutical substances.

In a screening study for the resolution of four isomers of tofisopam, Chirobiotic^TM^ T and TAG were tested, as well as polysaccharide and cyclodextrin-based columns [54]. Regardless of the mobile phase employed, no chiral separation was observed on the macrocyclic glycopeptide-based columns. A method applying Chirobiotic^TM^ T for the chiral resolution of quinolones was developed and described with mobile phases consisting of MeOH, MeCN, water, and TEA [55]. In addition to the chromatographic data, the supramolecular mechanism of the chiral recognition was established by a modeling study, where hydrogen bonds and π–π interactions were found to be the major forces in chiral separation. Enantiomeric resolution of antibacterial agents, primaquine, quinacrine, and tafenoquine was achieved with Chirobiotic^TM^ R using mobile phases consisting of MeOH, MeCN, water, and TEA [56]. The optimized method was partially validated. 

Sardella et al. [57] developed a simple RP-HPLC method for the enantioseparation of carnosine, employing Chirobiotic^TM^ T column. The observed U-shaped retention behavior was attributed to increased hydrophobic interactions in water-rich mobile phases and decreased solubility in MeOH-rich mobile phases. Later, the same group identified all interactions playing important roles in the chiral recognition process using molecular dynamic simulations [58]. 

The unusual dynamic behavior of the enantiomers of pyrroloquinolones was studied on a Nautilus-R column under RP conditions [59]. A phenomenological explanation was provided for the observed differences between the van Deemter plots of the enantiomers. Chirobiotic^TM^ T, TAG, R, and V were applied for screening to find the optimal CSP for the RP separation of pantoprazole enantiomers [60]. Chirobiotic^TM^ TAG was found to offer the best resolution. The optimized and validated method was applied for the quantitative determination of pantoprazole in commercially available dosage forms. The enantiomer elution order was studied by circular dichroism spectroscopy and quantum chemical approach. Chromatographic behaviors of some dipeptides (Leu–Leu, Gly–Leu, and Leu–Gly) focusing on the pH effects were investigated on Chirobiotic^TM^ T and R [61]. Adsorption of Leu–Leu and Leu–Gly was found to rely on the ion–ion association between the solute and selector, which was controlled largely by the ionic forms of the solute and, to a smaller degree, by the ionization state of the selector. 

The enantioselective retention mechanisms employing dipeptides as model compounds were investigated on ristocetin-A-based CSPs by Asnin et al. [62,63,64] under RP conditions. Effects of mobile phase composition on the separation of Ala–Ala, Leu–Leu, Gly–Leu, and Leu–Gly were studied on a Chirobiotic^TM^ R column [62]. The lipophilicity of the dipeptides was found to be a determining parameter in the description of their retention behavior, strongly affected by the MeOH content of the mobile phase. In a similar study, the dynamics of adsorption of Leu–Leu stereoisomers on a Chirobiotic^TM^ R column was explored using Gly–Gly for comparison [63]. The observed peculiarities of van Deemter plots were explained by the eddy dispersion, while the adsorption kinetics were found to have only secondary importance. A striking difference in the enantioselectivities of Chirobiotic^TM^ R and Nautilus-R was observed in the enantiomeric separations of Ala–Ala, Gly–Leu, Leu–Gly [64]. Results were interpreted on the basis of the differing anchoring methods used to immobilize the selector onto the silica support, but the contribution of the structural variations of the binding sites to the observed phenomena could not be excluded.

Subcritical fluid chromatography (SFC) with Chirobiotic^TM^ T column was used for the enantioseparation of Phe, Tyr, and Trp in a mobile phase system containing CO_2_, MeOH, and H_2_O [65]. The optimized method was validated and applied for the enantiomeric purity control of five food supplements. The chromatographic performance of Chirobiotic^TM^ T, TAG, and V2 was compared under SFC conditions using CO_2_ and MeOH as mobile phase constituents without additives [66]. Chemometric methods based on linear solvation energy relationships were applied for the characterization of the studied CSPs, allowing some insights into the retention mechanism and chiral recognition.

### 4.3. Enantioseparations Achieved with Macrocyclic Glycopeptides Bonded on Ultra-High-Performance Particles

As we briefly discussed in the introduction, reducing the size of the particles utilized for packing may offer the possibility for very fast enantioseparations. Efforts made on this resulted in the publication of several research papers, however, the number of commercially available columns packed with ultra-high-performance particles is still rather limited. Recent results obtained with macrocyclic antibiotic selectors are summarized in Table 5.

The chromatographic performances of CSPs based on teicoplanin, teicoplanin aglycon, vancomycin, cyclofructanes, and cyclodextrins bonded on superficially porous particles were studied for the separation of 60 pairs of enantiomers [16]. Varying the mobile phase conditions (RP, NP, PI) and CSPs, baseline separation could be achieved within a minute for all enantiomer pairs, but the studied CSPs were found to possess rather different kinetic profiles. Two types of sub-2 µm FP silica particles were employed as support materials for the preparation of CSPs based on vancomycin, teicoplanin, and teicoplanin aglycon [67]. Particles with a narrow size distribution were easier to pack, with reduced plate heights compared to polydisperse particles. The increased permeability of these columns allowed baseline separation for the enantiomers of twenty-three analytes (e.g., amino acids, β-blockers, heterocyclic compounds) in most cases under a minute. The applicability of teicoplanin-bonded sub-2 µm SP particles was demonstrated for the enantioseparation of six native amino acids under RP conditions [68]. Compared to earlier results using the same bonding method and the same mobile phase, shorter retention time, higher resolution, and improved selectivity were obtained.

Teicoplanin, CF-6, and quinine-based 2.7 µm SP particles were packed into 0.5 cm-long columns [69]. The sub-second chromatographic (HILIC, RP, and chiral) separations were analyzed from conceptual and practical aspects focusing on hardware considerations. The effect of the non-Gaussian dispersion on peak shapes in short tubings was found to be the most significant future challenge, which can be circumvented with techniques based on on-column injection and on-column detection systems. 

CSPs based on vancomycin, teicoplanin, cyclofructane, and hydroxypropyl-β-cyclodextrin bonded on 2.7 µm SP particles were evaluated for the separation of fluorinated and desfluorinated compound mixtures [70]. Better efficiencies were obtained with SP particle-based CSPs than with commercially available CSPs based on 5 µm FP particles. Commercially available vancomycin- and teicoplanin-based UHPLC columns (VancoShell, TeicoShell, AZYP, LLC, Arlington, TX, USA) were utilized for the separation of peptides [71]. In the tryptic peptide separations studied, competitive separation characteristics of the TeicoShell column with different selectivities were recorded compared to a commercial C18 phase when developing MS-compatible isocratic methods. Utilizing a CSP (NicoShell) containing a modified macrocyclic glycopeptide, an enantioselective method was developed for the resolution of nicotine and nornicotine [72]. The method was successfully applied for the determination of the enantiomeric ratio of nicotine in various tobacco products. This approach was further elaborated for the analysis of nicotine-related compounds [73]. Complementary separations were seen in several cases using macrocyclic antibiotic-based commercial columns (VancoShell, TeicoShell, NicoShell, AZYP, LLC, Arlington, TX, USA) offering the possibility for high-throughput investigations. 

Gasparrini et al. [74,75,76] studied teicoplanin-based CSPs prepared on silica particles of high efficiency, applying a bonding protocol to ensure zwitterionic character for the teicoplanin selector. In the first study, a teicoplanin-based CSP was prepared using sub-2 µm totally porous silica particles of narrow size distribution [74]. The kinetic performance of the columns of different lengths was systematically evaluated based on van Deemter plots using both chiral and achiral analytes. The zwitterionic character of the teicoplanin-based CSP was found to offer unique multipurpose properties in the separation of charged or neutral analytes under RP, NP, POM, HILIC, or SFC conditions. Applying the same bonding protocol (ensuring zwitterionic character for the teicoplanin selector), SP particles of 2 µm and 2.7 µm, and FP particles of 1.9 µm were prepared [75], and the kinetic profiles of the CSPs were characterized in a similar fashion as was reported earlier [74]. The columns packed with 2 µm SP particles exhibited excellent performance with a plate number of 300,000 N/m, predicting promising future applications. New CSPs based on sub-2 µm FP silica particles with narrow size distribution were prepared and employed for chiral and achiral separations [76]. In this study vancomycin- and teicoplanin-based CSPs were prepared in two different ways; specifically, applying a traditional ureidic linkage or ensuring zwitterionic character for the selectors. The zwitterionic CSPs were found to possess different selectivities, and they were applicable under RP, HILIC, and weak ion exchange conditions. 

Ultrafast chiral separations based on vancomycin (Vanco-FPP), and teicoplanin (Teico-FPP) bonded to FP silica particles of 1.9 µm were demonstrated to be effective as a second dimension for 2D chromatographic systems [77]. The developed multiple heart-cutting 2D-LC method proved to be an efficient tool for the analysis of enantiomeric compounds in multicomponent samples. A method applying a conventional HPLC system was developed for the separation of enantiomers of four intermediates and the pharmaceutical ingredient (API) of verubecestat, as an experimental drug of Alzheimer’s disease [78]. The application of commercial TeicoShell (4.6 × 150 mm and 4.6 × 100 mm) and NicoShell (4.6 × 150 mm) columns packed with 2.7 µm particles in isocratic RP mode provided a simple analytical procedure to achieve efficient separations.

A comprehensive study of 150 primary, secondary, and tertiary amines related to pharmacology and toxicology pointed out the importance of macrocyclic glycopeptide-based chiral selectors in the enantiomeric separation of amines [79]. Overall, baseline separation was obtained in 113 cases on the VancoShell column, and 110 racemic compounds on the NicoShell column in a multimodal fashion. The Edman degradation product (EDP) of vancomycin, as the newest vancomycin derivative selector was evaluated in the enantiomeric separation of 50 biologically active chiral compounds including stimulants, nonsteroidal anti-inflammatory drugs, pesticides, acidic, and basic APIs [80]. The separation characteristics of the new selector bonded on 2.7 µm SP particles were compared to results obtained with VancoShell, TeicoShell, and NicoShell columns applied in RP, NP, PI, and PO modes. For all CSPs, the PI was found to be the most successful chromatographic mode in the screening procedure, and the EDP CSP was shown to behave similarly to the TeicoShell column in the enantiomeric separation of amino acids, herbicides, and nonsteroidal anti-inflammatory drugs. New effective methods with several core-shell CSPs with selectors based on cyclofructan, macrocyclic glycopeptide, and quinine were utilized to separate 100 chiral pesticides using MS-compatible mobile phases [81]. Overall, all isomers of 74 pesticides were baseline-separated on at least one CSP, with analysis times less than 10 minutes in most cases. In the case of macrocyclic glycopeptide selectors, high enantiomeric selectivity was obtained for acidic pesticides using TeicoShell CSP, while the NicoShell and VancoShell CSPs were efficient in the chiral separation of pesticides with amine functionalities. Enantiomeric separation of twelve new chiral azole compounds was investigated with six different CSPs, four of them possessing macrocyclic glycopeptide selector (NicoShell, TagShell, TeicoShell, VancoShell, AZYP, LLC, Arlington, TX, USA) [82]. Regarding mobile phase composition, the application of RP mode resulted in better enantiomeric separations of azole compounds on macrocyclic glycopeptide CSPs than using NP or PO conditions. 

The relative merits of 2.7 µm SP particles for enantiomeric separations of 100 chiral analytes were compared to CSPs based on 5 µm FP particles [83]. Applying SFC conditions and VancoShell, TeicoShell, NicoShell, or a cyclofructane-based column, all separations were achieved within 10 min. The advantageous properties of SP particles operated at high flow rates were supported by the obtained van Deemter plots. Nine CSPs (including VancoShell, TeicoShell, and NicoShell) with different surface chemistries were studied to gather information on the role of water in chiral SFC [84]. The affinity of the CSPs for water was examined with reference to bare silica with marked improvements in efficiency found with hydrophilic CSPs. On the basis of the previously observed phenomenon [84], Armstrong et al. [85] suggested the application of azeotropic ethanol (“190 proof”) for SFC separations. Investigating eight chiral columns (three of them with macrocyclic antibiotic selector), the advantageous properties of “190 proof” ethanol as a polar co-solvent was demonstrated. Namely, better chromatographic efficiencies and often shorter retention times were obtained compared to methanol, especially with polar CSPs. The enantioseparation of biologically active and structurally diverse chiral compounds was investigated employing VancoShell and TeicoShell columns under SFC conditions [86]. The influence of mobile phase conditions on retention and enantioselectivity was examined, and complementary behavior of the macrocyclic antibiotic-based columns was confirmed.

## Figures and Tables

**Table 1:** Summary of the physico-chemical properties of macrocyclic antibiotics applied as chiral selectors [13].

Properties		Ansamycins			Glycopeptides				
		Rifampicin	Rifamycin B	Rifamycin SV	Avoparcin	Teicoplanin A_2–2_	Teicoplanin A-40,926	Teicoplanin MDL 63,246	Dalbavancin
Molecular weight		823	756	698	α = 1908 β = 1943	1877	B_0_ = 1732 B_1_ = 1718	1789	1817
Hydrophobic tail		0	0	0	0	1	1	2	2
Number of …	asymmetric centers	9	9	9	32	23	B_0_ = 19 B_1_ = 18	18	18
	macrocycles	1	1	1	3	4	4	4	4
	aromatic rings	2	2	2	7	7	7	7	7
	sugar moieties	0	0	0	5	3	2	2	2
	hydroxy groups	5	5	5	16	14	11	12	11
	primary amines	0	0	0	2	1	0	0	0
	secondary amines	0	0	0	1	0	1	1	1
	amido groups	1	1	1	6	8	7	8	8
	carboxylic groups	1	1	0	1	1	2	0	1
	methoxy groups	1	1	1	0	0	0	0	0
	methyl esters	1	1	1	0	0	0	0	0
Produced by		*Amycolatopsis rifamycinica*	*Nocardia mediterranei*	*Nocardia mediterranei*	*Streptomyces candidus*	*Actinoplanes teichomycetius*	*Nonomuraea ATCC 39727*	Synthetic compound	Synthetic compound

**Table 2:** Summary of the physico-chemical properties of macrocyclic antibiotics applied as chiral selectors [13].

Properties		Glycopeptides						Polypeptides
		Teicoplanin Aglycone	Ristomycin Ristocetin A	Vancomycin	Nor-Vancomycin	Eremomycin	Balhimycin	Thiostrepton
Molecular weight		1197	2066	1449	1435	1558	1446	1665
Hydrophobic tail		0	0	0	0	0	0	0
Number of …	asymmetric centers	8	38	18	18	22	17	17
	macrocycles	4	4	3	3	3	3	2
	aromatic rings	7	7	5	5	5	5	1
	sugar moieties	0	6	2	2	3	2	0
	hydroxy groups	7	21	9	9	9	8	5
	primary amines	1	2	1	2	3	1	0
	secondary amines	0	0	1	0	0	1	1
	amido groups	6	6	7	7	7	7	11
	carboxylic groups	1	0	1	1	1	1	0
	methoxy groups	0	0	0	0	0	0	0
	methyl esters	0	1	0	0	0	0	0
Produced by		*Synthetic compound*	*Nocardia lurida*	*Streptomyces orientalis*	*Streptomyces orientalis*	*Amycolatopsis orientalis*	*Amycolatopsis balhimycina*	*Streptomyces azureus*

**Table 3:** Enantioseparation of stereoisomers of different analytes on vancomycin- and vancomycin-related analog-based CSPs ^1^.

Analytes	Selectors	Column’s Trade Mark	The Most Effective Mobile Phases (*v/v/v*)	Mode	Reference
trantinterol	Vancomycin	Chirobiotic V	MeOH/MeCN/AcOH/NH_4_OH 80/20/0.02/0.01 or 60/40/0.02/0.01	PIM	[26]
amphetamine, metamphetamine	Vancomycin	Chirobiotic V Chirobiotic V2	MeOH/AcOH/NH_4_OH 100/0.1/0.02	PIM	[27]
amphetamine, metamphetamine, methylenedioxyamphetamine, methorphan, methylenedioxymetamphetamine, ephedrine, pseudoephedrine	Vancomycin	Chirobiotic V2	MeOH/0.04% NH_4_TFA	PIM	[28]
ketoprofen	Vancomycin	chiral mobile phase additive	0.05 M KH_2_PO_4_ (pH 6.0)/2-propanol 50/50/	RPM	[29]
amlodipin, atropine, baclofen, ibuprofen, mandelic acid, Phe	Vancomycin degradation product	tailor-made	0.1% NH_4_TFA in MeOH aq. TEAA (pH 6.5)/MeOH (85/15) 20 mM aq. sodium citrate (pH 6.3)/THF	PIM RPM RPM	[30]
metoprolol, pindolol, alprenolol, oxprenolol, labetolol, atenolol Trp, Phe, DOPA, Met, Glu, Ala, Nva, Val, Lys, arg, Ser	immobilized mixed Eremomycin and Vancomycin on silica	tailor-made	0.1% aq.TEAA (pH 4.5)/MeOH/MeCN (5/20/75) MeCN/aq. AcOH (97/3)	RPM	[31]
Β-blockers: nadolol, atenolol, metoprolol, alprenolol, oxprenolol, pindolol	Vancomycin on gold nanoparticles	tailor-made	25 mM potassium phosphate (pH 4)/MeCN (96/4) 25 or 50 mM ammonium acetate (pH 4)/MeCN (96/4)	RPM	[32]
mandelic acid, propranolol	Vancomycin	tailor-made	*n*-heptane/2-propanol (90/10) containing 0.4% TFA	NPM	[33]
chiral xanthone derivatives	Vancomycin, Teicoplanin, Teicoplanin aglycone, Ristocetin A	Chirobiotic V Chirobiotic T Chirobiotic TAG Chirobiotic R	*n*-hexane(EtOH or *n*-hexane/2-PrOH aq. TEAA (pH 4.2)/MeOH; NH_4_OAc (pH 6)/MeOH 100% MeOH, 100% EtOH or 100% 2-PrOH MeOH(AcOH/TEA)	NPM RPM POM PIM	[34]
nine aromatic hydroxy acids	Eremomycin Ristomycin Teicoplanin	Nautilus-E Nautilus-R Chirobiotic T	aq. NH_4_OAc (pH 3.3–6.3)/EtOH (60/40)	RPM	[35]
ketoprofen, flurbiprofen, suprofen, carprofen, ibuprofen, warfarin	Clindamycin phosphate (CLIP) and Erythromycin incorporated to zircona hybrid monolith	CLIP-ZHM ERY-ZHM	MeOH/MeCN (20/80) containing 300 mM AcOH and 10 mM TEA	CEC	[36]
atenolol, chlorphenamine, esmelol, nefopam, propranolol	azithro-mycin lactobionate, clindamycin phosphate	tailor-made	freshly dissolved azithro-mycin lactobionate and clindamycin phosphate in phosphate buffer (20 mM) adjusted to specific pH with sodium hydroxide	CEC	[37]

^1^ RPM, reversed-phase mode; NPM, normal phase mode; PIM, polar-ionic mode; POM, polar-organic mode; CEC, capillary electrochromatography.

**Table 4:** Enantioseparation of stereoisomers of different analytes on teicoplanin, teicoplanin aglycon, vancomycin and on ristocetin A-based CSPs.

Group of Racemates	Racemates	Selector	Column’s Trade Mark	The Most Effective Mobile Phase (*v*/*v*/*v*)	Mode	Reference
Amino acids amino acid analogs	phenylisoserine derivatives	Teicoplanin Teicoplanin aglycone, Vancomycin, Vancomycin aglycone	Chirobiotic T Chirobiotic TAG Chirobiotic V Chirobiotic VAG	0.1% TEAA (pH 4.1)/MeOH (50/50)	RPM	[39]
	Met, Cys, homo-Cys	Teicoplanin Teicoplanin aglycone	Chirobiotic T Chirobiotic TAG	25 mM aq. phosphate buffer/1 mM aq. octanesulfonic acid (pH 2.7)/MeCN/MeOH (94/3/3)	RPM	[40,41,42]
	therapeutic peptides	Teicoplanin, Vancomycin	Chirobiotic T Chirobiotic V	20 mM aq. NH_4_OAc (pH 4.1)/MeCN (5/95) 0.1% aq. TEAA/MeOH (90/10)	HILIC RPM	[43]
	carbocyclic β-amino acids possessing limonene skeleton	Teicoplanin Teicoplanin aglycone Ristocetin A	Chirobiotic T Chirobiotic TAG Chirobiotic R	MeOH/AcOH/TEA (100/0.01/0.01) and (100/0.1/0.1) 0.1% aq. TEAA/MeOH (90/10)	PIM RPM	[44]
	Phe	Teicoplanin Ristocetin A	Chirobiotic T Chirobiotic R	MeCN/H_2_O (75/25) MeCN/H_2_O (60/40)	RPM	[45]
Drugs	ofloxacin	Teicoplanin Teicoplanin aglycon Ristocetin A	Chirobiotic T Chirobiotic TAG Chirobiotic R	0.45% aq. TEAA (pH 3.6)/EtOH (20/80) 0.45% aq. TEAA (pH 3.6)/EtOH (80/20)	RPM	[46]
	epimeric mixtures of fortimicin aminoglycosides	Teicoplanin	Chirobiotic T	10 mM ammonium formate/MeOH	PIM	[47]
	citalopram analogs	Teicoplanin Teicoplanin aglycone, Vancomycin, Ristocetin A	Chirobiotic T Chirobiotic TAG Chirobiotic V Chirobiotic V2 Chirobiotic R	0.1% aq. TEAA (pH 4.1)/MeOH 0.1% aq. TEAA	RPM PIM	[48]
	modafanil	Teicoplanin	Chirobiotic T	MeOH/TEA (100/0.05)	PIM	[49]
Drugs	ibuprofen, carboxyibuprofen, 2-hydroxy ibuprofen, chloramphenicol, ifosfamide, indoprofen, ketoprofen, naproxen, praziquantel	Teicoplanin	Chirobiotic T	aq. 10 mM NH_4_OAc (pH 4.2)/MeOH (70/30)	RPM	[50]
Drugs Peptides	mandelic acid, vanylmandelic acid, phenyllactic acid	Teicoplanin + ionic liquids	Chirobiotic T	MeOH/H_2_O + borneol or fenchol-based ionic liquids	RPM	[51,52]
	albuterol	Teicoplanin aglycon Eremomycin	Chirobiotic TAG Nautilus-E	MeOH/MeCN/TEA/AcOH (90/10/0.05/0.05) MeOH/MeCN/TEA/AcOH (80/20/0.075/0.025)	PIM	[53]
	tofisopam	Teicoplanin Teicoplanin aglycone,	Chirobiotic T Chirobiotic TAG	0.1% TEAA (pH 4.1)/MeOH	RPM	[54]
	primaquine, tafenoquine, flumequine, lomefloxacine, ofloxacin, qunacrine	Teicoplanin	Chirobiotic T	MeOH/MeCN/water/TEA (70/10/20/0.01); (60/30/10/0.1) and (50/30/20/01)	PIM	[55]
	primaquine, quinacrine, tafenoquine	Ristocetin A	Chirobiotic R	MeOH/MeCN/water/TEA (70/10/20/0.1); (60/30/10/0.1)	PIM	[56]
	carnosine	Teicoplanin	Chirobiotic T	aq. formic acid/MeOH (80/20–20/80), pH_a_ 3.1–3.8 20 mM ammonium formate/MeOH (40/60), pH_a_ 4.5	RPM	[57,58]
	pyrroloquinolo-ne analogs	Ristocetin A	Nautilus-R	water/MeCN (65/35)	RPM	[59]
	pantoprazole	Teicoplanin aglycon Teicoplanin Ristocetin A Vancomycin	Chirobiotic TAG Chirobiotic T Chirobiotic R Chirobiotic V	aq. 20 mM NH_4_OAc/MeOH (40/60)	RPM	[60]
	Leu–Leu, Gly–Leu, Leu–Gly	Teicoplanin Ristocetin A	Chirobiotic T Chirobiotic R	aq. 0.097 M AcOH + 0.003 M NH_4_OAc (pH 3.85)/MeCN aq. 0.003 M AcOH + 0.097 M NH_4_OAc (pH 6.80)/MeCN	RPM	[61]
Peptides Amino acids	Ala–Ala, Leu–Leu, Gly–Leu, Leu–Gly	Ristocetin A	Chirobiotic R	aq. 0.0002 M NH_4_OAc/MeOH (100/0–10/90)	RPM	[62]
	Leu–Leu, Gly–Gly	Ristocetin A	Chirobiotic R	aq. 0.0002 M NH_4_OAc/MeOH (90/10)	RPM	[63]
	Ala–Ala, Gly–Leu, Leu–Gly	Ristocetin A Ristocetin A	Chirobiotic R Nautilus-R	aq. 100 mM NH_4_OAc/MeOH (60/40)	RPM	[64]
	Phe, Tyr, Trp	Teicoplanin	Chirobiotic T2	CO_2_/(MeOH/water) 60/(90/10)	SFC	[65]
Miscellenous	67 racemates: amino acids, β-blockers, profens, pesticides, etc.	Teicoplanin Teicoplanin aglycon Vancomycin	Chirobiotic T Chirobiotic TAG Chirobiotic V2	CO_2_/MeOH 90/10; CO_2_/MeOH (90/10) + 0.1% formic acid or diethylamine in CO_2_ *n*-heptane/EtOH (90/10)	SFC NPM	[66]

**Table 5:** Ultra-high-performance liquid chromatographic enantioseparations of different analytes on macrocyclic glycopeptide selectors immobilized on sub-2 µm superficially porous (core-shell) and fully porous particles.

Racemates	Selector	Column Characteristics Trade Mark Particle Size	The Most Effective Mobile Phase *(v/v/v)*	Mode	Reference
60 pairs of enantiomers of: amino acids, peptides, primary amines, β-blockers, thalidomide, nicardipine, proglumide, coumachlor, warfarin, mianserin, etc.	Teicoplanin Teicoplanin aglycone, Vancomycin	TeicoShell, SPP, 2.7-μm TagShell, SPP, 2.7-μm VancoShell, SPP, 2.7-μm	water/MeOH (99/1–10/90) aq. 1.0% TEAA (pH 4.1)/MeCN (80/20) MeOH/AcOH/TEA (100/0.15/0.05)	RPM NPM PIM	[16]
amino acids, β-blockers, oxazolidinones, mandelic acid, coumachlor, proglumide, thalidomide, warfarin, mianserin	Teicoplanin Teicoplanin aglycone, Vancomycin	Titan-T, FPP-NPSD, 1.9-μm Titan-TAG, FPP-NPSD, 1.9-μm Titan-V, FPP-NPSD, 1.9-μm	*n*-heptane/EtOH (80/20) water/MeOH (40/60–20/80) 0.1% TEAA (pH 4.1)/MeCN (80/20) 100% MeOH MeOH/MeCN/AcOH/TEA (45/55/0.3/0.2) and (40/60/0.3/0.2) CO_2_/MeOH/TFA/TEA (71/29/0.1/0.1) or (60/40/0.1/0.1)	NPM RPM POM PIM SFC	[67]
Met, Val, Leu, Ala, Nval, Nleu	Teicoplanin	SPP, sub-2-μm	EtOH/water (80/20) and (90/10) MeOH/water (90/10)	RPM	[68]
DNPyr–Leu, DNPyr–Nval, *N*-acetyl-Ala, *N*-3,5-DNB–Leu, 4-methyl-5-phenyl-2-oxazolidinone	Teicoplanin	SPP, 2.7-μm	100% MeOH aq. 20 mM NH_4_HCOO/MeOH (40/60) or (30/70) aq. 5 mM NH_4_HCOO/MeOH/MeCN (40//40/20)	POM RPM PIM	[69]
fluorinated, desfluorinated analytes: ofloxacin, cipro-floxacin, ezitimibe, paro-xetine, voriconazole, aprepitant, atorvastatin	Teicoplanin Vancomycin	TeicoShell, SPP, 2.7-μm VancoShell, SPP, 2.7-μm	MeOH/MeCN/TFA/TEA (10/90/0.3/0.2) MeOH/MeCN/TFA/TEA (50/958/0.3/0.2)	PIM	[70]
enkephalin, bradykinin, vasopressin, LHRH peptides triptic digest of equine apomyoglobin	Teicoplanin Teicoplanin aglycone	TeicoShell, SPP, 2.7-μm VancoShell, SPP, 2.7-μm	2.5–50 mM NH_4_HCOO (pH 3.2)/MeCN/ (65/35, 30/70) 50 mM NH_4_HCOO (pH 3.2)/MeOH (50/50) 50 mM NH_4_HCOO (pH 3.2)/THF (90/10) or (80/20)	RPM	[71]
nicotine	modified teicoplanin	NicoShell, SPP, 2.7-μm	0.1% ammoniumtrifluoro acetate in MeOH	RPM	[72]
tobacco alkaloids synthetic tobacco deri-vatives tobacco metabolites (E/Z)-tobacco-nitrosamines	Teicoplanin Teicoplanin (modified) Vancomycin	TeicoShell, SPP, 2.7-μm NicoShell, SPP, 2.7-μm VancoShell, SPP, 2.7-μm	0.025–0.5 wt% HCOONH_4_ in MeOH MeOH/MeCN/AcOH/NH_4_OH aq. 16 mM HCOONH_4_/MeOH aq. 16 mM HCOONH_4_/EtOH aq. 16 mM HCOONH_4_/MeCN	PIM POM RPM	[73]
*N*-protected amino acids, α-aryloxy acids, herbicides, anti-inflammantory agents	Teicoplanin	zwitterionic phases UHPC-Titan120-Tzwitt, FPP 1.9-μm	20 mM aq. NH_4_OAc/MeOH (15/85) 20 mM aq. NH_4_OAc/MeCN (15/85) MeOH/MeCN/AcOH/TEA (40/60/0.055/0.03) *n*-hexane/EtOH (70/30)	RPM HILIC PIM NPM	[74]
2-(4-chloro-phenoxy)-propionic acid d,l-proglumide, danzyl-d,l-Met, Fmoc-d,l-Glu, Z-d,l-Met	Teicoplanin	zwitterionic phases UHPC-Halo-Tzwitt, SPP-2.0-μm UHPC-Halo-Tzwitt,, SPP-2.7-μm UHPC-Titan-Tzwitt, FPP-1.9-μm	aq. 20 mM HCOONH_4_ (pH 7.5)/MeCN (15/85)	HILIC	[75]
haloxyfop, ketolarac, ketoprofen, indoprofen, flunoxaprofen, naproxen, suprofen, ibuprofen, Fmoc-, Boc-, Z-, danzyl-amino acids	Teicoplanin Vancomycin	zwitterionic phases UHPCTitan120-Tzwitt, FPP-1.9-μm UHPC-Titan120-Vzwitt, FPP-1.9-μm anion exchangers UHPC-Titan120-T_COOH_, FPP-1.9-μm UHPC- Titan120-V_COOH_, FPP-1.9-μm	aq. 10 mM HCOONH_4_ (pH 6.5)/MeOH (15/85) aq. 20 mM HCOONH_4_ (pH 6.5)/MeOH (15/85) aq. 15 mM CH_3_OONH_4_/MeCN 15/85 aq. 15 mM CH_3_OONH_4_ (pH 5.5)/MeCN (40/60) aq. 15 mM CH_3_OONH_4_ (pH 5.5)/MeOH (40/60)	RPM HILIC	[76]
4-, 6-, 7-, 8- and 10-hydroxywarfarins, 4-phenylacetic acid, 2-, 3-, 4-, 6-fluorophenyl-acetic acid, 2,4-, 3,5-difluoro-phenylacetic acid	Teicoplanin Vancomycin	2D-LC Teico-FPP, 1.9-μm Vanco-FPP, 1.9-μm	aq. 5% H_3_PO_4_/MeCN 95/5	RPM	[77]
intermediates of verubecestat synthesis	Teicoplanin Teicoplanin (modified)	TeicoShell, SPP, 2.7-μm NicoShell, SPP, 2.7-μm	aq. 0.1% H_3_PO_4_/MeCN (70/30) aq. 1.0–2.0% TEAA/MeOH (70/30) aq. 1.0–2.0% TEAA/MeCN (40/60)	RPM	[78]
150 primary-, secondary- and tertiary-amines	Vancomycin Teicoplanin (modified)	VancoShell, SPP, 2.7-μm NicoShell, SPP, 2.7-μm	aq. NH_4_TFA/MeOH; aq. HCOONH_4_/MeOH; aq. HCOONH_4_/MeCN; aq. HCOONH_4_/EtOH; MeOH/AcOH/NH_4_OH; MeOH/MeCN/AcOH/TEA;	RPM PIM	[79]
amines, amino acids and derivatives; non-steroidal anti-inflammantory drugs, pesticides, nicotine and metabolites	Vancomycin (EDP) Vancomycin Teicoplanin Teicoplanin (modified)	EDP, SPP, 2.7-μm VancoShell, SPP, 2.7-μm TeicoShell, SPP, 2.7-μm NicoShell, SPP, 2.7-μm	0.1 wt% HCOONH_4_ in MeOH MeOH/MeCN/AcOH/TEA (40/60/0.3/0.2) aq. 16 mM HCOONH_4_ (pH 3.6) (30/70) *n*-hexane/EtOH/TFA/TEA (70/30/0.3/0.2)	PIM POM RPM NPM	[80]
100 pesticides: pyrethroids, fungicides, organophosphates, acylanilides, herbicides, rodenticides, etc.	Teicoplanin Vancomycin Teicoplanin (modified)	TeicoShell, SPP, 2.7-μm VancoShell, SPP, 2.7-μm NicoShell, SPP, 2.7-μm	0.025–0.5 wt% HCOONH_4_ in MeOH aq. 16 mM HCOONH_4_ (pH 3.6)/MeOH (90/10–30/70) aq. 16 mM HCOONH_4_ (pH 3.6)/MeCN (90/10–20/80) *n*-hexane/EtOH/TFA/TEA (70/30/0.3/0.2)	PIM RPM NPM	[81]
azole compounds: oxazols, thiazols	Teicoplanin Teicoplanin aglycon Vancomycin Teicoplanin (modified)	TeicoShell, SPP, 2.7-μm TagShell, SPP, 2.7-μm VancoShell, SPP, 2.7-μm NicoShell, SPP, 2.7-μm	aq. 20 mM HCOONH_4_ (pH 3–4)/MeOH (85/15–50/50) aq. 16 mM HCOONH_4_ (pH 3–6)/MeCN (85/15–20/80) MeOH/EtOH (25/75; 50/50)	RPM POM	[82]
100 chiral analytes: amines, derivatized amino acids, nicotine and metabolites, β-blockers, pesticides, drugs etc.,	Teicoplanin Vancomycin Teicoplanin (modified)	TeicoShell, SPP, 2.7-μm VancoShell, SPP, 2.7-μm NicoShell, SPP, 2.7-μm	0.1% TEA in MeOH/CO_2_ (25/75) 0.1 wt% HCOONH_4_ in MeOH/CO_2_ (25/75) 0.1% TFA in MEOH/CO_2_ (25/75) 0.1% TEA + 0.1–0.3% TFA in MeOH/CO_2_ (25/75)	SFC	[83]
cis-4,5-diphenyl-2-oxazo-lidinone, 2-(4-chloro-phenoxy)propionic acid, fluoxetine, nicardipine, bupivacaine, roglumide	Teicoplanin Vancomycin Teicoplanin (modified)	TeicoShell, SPP, 2.7-μm VancoShell, SPP, 2.7-μm NicoShell, SPP, 2.7-μm	MeOH/CO_2_ (5/95) 0.1% TEA + 0.1% TFA in MeOH/CO_2_ (20/80; 15/85) 0.1% TEA + 0.1 % TFA+ 6% water in MeOH/CO_2_ (20/80) 0.1 wt% HCOONH_4_ in MeOH/CO_2_ (20/80)	SFC	[84]
phytoalexins, substituted tryptophanes, ketamine derivatives	Teicoplanin Vancomycin	TeicoShell, SPP, 2.7-μm VancoShell, SPP, 2.7-μm	0.05–0.1% DEA, TEA, TFA or 2-propylamine in MeOH, EtOH, 2-PrOH or 1-PrOH/CO_2_ (20/80; 60/40; 5/95)	SFC	[85]
cis-4,5-diphenyl-2-oxazolidinone, chlorthalidone, 5,5-diphenyl-4-benzyl-2-oxazolidinone, nicotine, bupivacaine, prilocaine, tranylcypromine, amphetamine, venlafaxine, tryptophan, 1,2,2-triphenylethylamine, 2-chloro-indan-1-ylamine, disopyramide, tetramisole, fenoprofen	Teicoplanin Vancomycin Teicoplanin (modified)	TeicoShell, SPP, 2.7-μm VancoShell, SPP, 2.7-μm NicoShell, SPP, 2.7-μm	“190” EtOH/CO_2_ (20/80; 25/75) 0.1% TEA in “190” EtOH/CO_2_ (40/60; 20/80) 0.1% TEA + 0.1% TFA in “190” EtOH/CO_2_ (20/80; 25/75) 0.2% TEA + 0.3% TFA in “190” EtOH/CO_2_ (25/75; 20/80; 15/85)	SFC	[86]

## Data Availability

Not applicable.

## Sample Availability

Samples of the compounds are not available from the authors.
